# Underserved ‘Deep End’ populations: a critical analysis addressing the power imbalance in research

**DOI:** 10.3399/bjgp23X733461

**Published:** 2023-06-30

**Authors:** Caroline Mitchell, Kate Fryer, Nicola Guess, Habiba Aminu, Ben Jackson, Anna Gordon, Josephine Reynolds, Qizhi Huang, Shamanthi Jayasooriya, Rebecca Mawson, Tom Lawy, Emma Linton, Janet Brown

**Affiliations:** Academic Unit of Primary Medical Care, Faculty of Medicine, Dentistry and Health, University of Sheffield, Sheffield.; Academic Unit of Primary Medical Care, Faculty of Medicine, Dentistry and Health, University of Sheffield, Sheffield.; Nuffield Department of Primary Care Health Sciences, Medical Sciences Division, University of Oxford, Oxford.; Academic Unit of Primary Medical Care, Faculty of Medicine, Dentistry and Health, University of Sheffield, Sheffield.; Academic Unit of Primary Medical Care, Faculty of Medicine, Dentistry and Health, University of Sheffield, Sheffield.; Elizabeth Blackwell Institute, University of Bristol, Bristol.; Academic Unit of Primary Medical Care, Faculty of Medicine, Dentistry and Health, University of Sheffield, Sheffield.; Academic Unit of Primary Medical Care, Faculty of Medicine, Dentistry and Health, University of Sheffield, Sheffield.; Academic Unit of Primary Medical Care, Faculty of Medicine, Dentistry and Health, University of Sheffield, Sheffield.; Academic Unit of Primary Medical Care, Faculty of Medicine, Dentistry and Health, University of Sheffield, Sheffield.; Academic Unit of Primary Medical Care, Faculty of Medicine, Dentistry and Health, University of Sheffield, Sheffield.; Academic Unit of Primary Medical Care, Faculty of Medicine, Dentistry and Health, University of Sheffield, Sheffield.; Department of Oncology and Metabolism, University of Sheffield, Sheffield.

## THE PROBLEM

Social inequalities are driven by power, income, and wealth, and shape health inequalities. The ‘inverse care law’ has enduring relevance to UK primary care. Underserved groups, including those living in poverty and those from ethnic minorities, spend more years with chronic conditions, have worse health outcomes, and poorer access to health care.^1^ It is self-evident that clinical research should generate results that are generalisable to the whole population.^2^ So why is ‘inverse representation’ in research the norm? The sociodemographic characteristics of participants in recent clinical trials suggest a mismatch with the representation of underserved populations, especially ethnic minorities.^3^ Inverse representation in research may be driven by discriminatory exclusion criteria that limit participation. This article focuses on researcher power, whereby researchers exercise control and influence inclusion in research, and suggests a theory-driven, empowering participatory approach to widen representation of underserved populations.

Commissioners of research, universities, and organisations that support recruitment may have prioritised efficiency (easier recruitment and lower attrition) over rigour (generalisable, representative sampling) and likelihood of implementation in all settings. Incorporation of flawed clinical trial evidence into clinical guidelines could widen health inequalities by shifting resources towards those interventions that work in populations at the lowest risk of poor outcomes. The prevalence of type 2 diabetes mellitus (T2DM) is significantly higher in ethnic minority and socioeconomically deprived populations; however, research that underpins a group education intervention (DESMOND) for people with T2DM recruited mostly White British people (94%) and did not report deprivation data.^4^ Referral activity to group education sessions for people with T2DM is incentivised by the UK GP Quality and Outcomes Framework, but uptake of these sessions in areas of high socioeconomic deprivation and among ethnic minorities is poor.^5^^,^^6^

Participation in clinical research has benefits for patients; for example, cancer outcomes are better in patients who participate in clinical trials.^7^ Interestingly, exposure of discrimination (by race, income, and sexual orientation) during recruitment to early clinical trials for HIV treatment galvanised excluded communities to demand fair access to trial participation.^8^ A powerful counterargument to participation in research for underserved groups includes the shameful, unethical abuse of power by researchers in a catalogue of historical injustices, such as the Tuskegee syphilis cohort study.^9^ Understandably there may be a lack of trust in public and private institutions in the UK that have a legacy of structural racism, classism, homophobia, transphobia, disablism, and colonialism. Recent migrant populations may have additional concerns related to coercive healthcare practices in their country of origin.^10^

Primary care researchers usually work within formal research delivery structures, such as the National Institute for Health and Care Research (NIHR) GP clinical research networks (CRNs) in England. Research demonstrates significant geographical variation in research activity across CRNs with disproportionately low recruitment in areas with a higher prevalence of chronic health conditions, perhaps reflecting the barriers to participation for underserved groups highlighted in the NIHR ‘INCLUDE’ guidance.^11^^,^^12^ These barriers included a lack of communication between research teams and participant groups, studies that exclude by design (for example, fail to recognise differential health literacy), a mismatch between researcher and participant agendas, and a lack of trust. One of four goals to increase inclusive representation in research was to build long-term relationships with underserved groups.

## THE DEEP END RESEARCH ALLIANCE IN YORKSHIRE-HUMBER (DERA)

The ‘Deep End’ (DE) movement originated in Scotland, and DE Projects address the inverse care law through networks of general practices working collaboratively to address health inequalities. In 2016, DE stakeholders in Yorkshire and the Humber suggested the ‘WEAR — Workforce, Education, Advocacy, Research’ — framework to prioritise and coordinate actions to address primary healthcare disparities.^13^

A collaborative group of academics and general practices in Sheffield (DERA) subsequently obtained funding to form a new DE-CRN and a patient and public involvement group (DE-PPI) to undertake research with underserved populations. The nine DE-CRN practices are situated in the most deprived areas of the UK by Index of Multiple Deprivation (IMD ≥40) and serve >68 000 ethnically diverse patient populations and homeless persons.

From the outset we embedded an ethos of ‘practitioner–patient–researcher partnership’ in the DE-CRN/-PPI groups, with regular meetings where researchers could share and shape research proposals with patients, primary care practitioners, and managers. Unfortunately, it became apparent that the machinery of UK research, from inception to delivery, favoured recruitment of health literate, ‘research-ready’ participants. Neither the DE-CRN practices nor their patients were ‘research ready’. The majority of NIHR portfolio studies that DE-CRN received were impossible to recruit to as the study design and recruitment materials ignored health literacy, were culturally incompetent in their approach, and none provided access to funded interpreters. We had to pre-vet presentations aimed at our combined PPI/practitioner meetings, as the researchers lacked lay communication skills.

Cognisant of reinforcing public mistrust through tokenistic PPI and a mismatch between real-world DE clinical practice and research realities, our DE-CRN, PPI group, and academic tripartite collaboration started a journey toward inclusive, co-created research with underserved communities and their practitioners.^14^ We trained researchers and students in the participatory research methods and skills necessary to work in partnership with underserved communities.^15^ We undertook eight qualitative research studies that recruited 118 participants from underserved groups (2017‒2021), conducted a survey in a migrant camp in Greece, and recruited participants to a clinical trial seeking participants from socioeconomically deprived populations.

## POWER SHARING AND CO-CREATION OF RESEARCH: AN ADAPTATION OF ARNSTEIN’S LADDER OF PARTICIPATION

Sherry Arnstein’s seminal paper, *‘A Ladder of Citizen Participation‘*, critiques the ‘democratic’ process by which communities influence the services they receive.^16^ She highlighted tokenistic approaches that valued ‘public relations’ over authentic community engagement. Arnstein’s central argument is that citizen participation requires the redistribution of power. Analogous barriers in academia to inclusive research might include researcher gender, technical skills, sexuality, race, and/or class-based positionality driving reticence about power sharing outside of universities. From the community perspective, barriers might include a lack of resources and research-specific abilities, and the difficulties of organising a representative and accountable PPI group within communities that are more used to alienation from institutions. Arnstein did not envisage that the ‘ladder’ would offer discreet solutions to power imbalance; her work highlighted that participation by all stakeholders requires effort, commitment, time, and trust.

**Figure 1.**
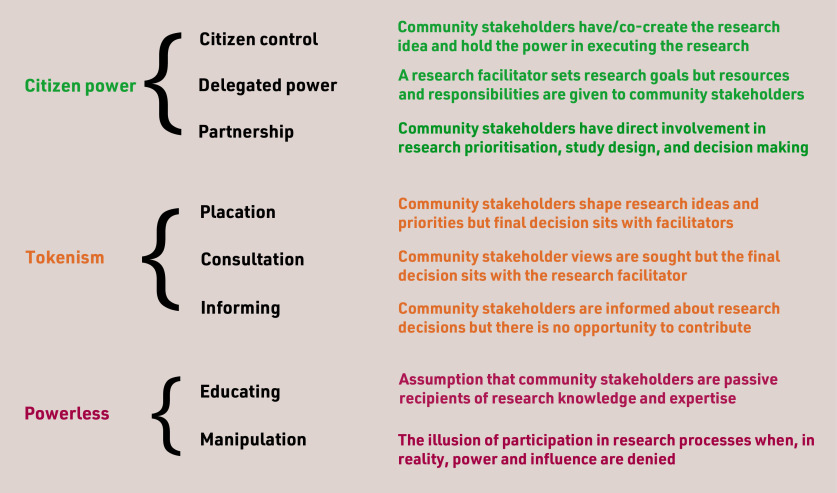
*Steps to inclusive research based on Arnstein’s* ‘A Ladder of Citizen Participation’*.^16^*

We have adapted Arnstein’s ‘Ladder of Citizen Participation’ (Figure 1) to represent some of the issues involved in the rebalancing of power between the researcher and the researched. Using ‘traffic light’ schemata we describe steps towards inclusive research that challenge the researcher power paradigm and could empower community members to shape the whole research process.

The following two DE-CRN case studies illustrate how rebalancing power and resources between researchers, patients, and community groups can build trust and connections to support research with and by underserved groups.

### Research with homeless women to explore their experiences of perinatal care: ‘partnership’

Women who are homeless experience a range of psychosocial challenges that influence access to care. These challenges include low health literacy and a high prevalence of mental illness and/or substance use. Despite high fertility rates and poor maternofoetal outcomes, there is little research about their experiences of perinatal care. Anna Gordon had embedded herself in the culture of an organisation serving homeless persons as a volunteer. The research idea came from conversations with women and charity workers, with further advice sought at the stages of formulation of a research question and study design. The standard format of patient research information sheets was unsuitable for people with poor health literacy. A patient information leaflet was co-produced using visual prompts to support informed consent. Building trust and taking a non-judgemental stance were important as the women described stigmatising experiences within healthcare settings. Results were shared in tailored lay, clinical, and academic formats to the service users, charity workers, perinatal NHS, and public health practitioners.^17^

### A partnership approach to develop prostate cancer research with people from a Black African Caribbean community: ‘delegated power’

Prostate cancer affects one in four black men and occurs at a younger age compared with their white counterparts. Black men are also underrepresented in clinical trials.^18^ With a grant shared between the university and our community partner (SACMHA Health and Social Care), we set up three participatory workshops co-facilitated by community researchers.

SACMHA identified two female volunteers as potential co-researchers. The women were not experienced in research. Our community researchers advised us on how to conduct facilitated discussions and fed back on the initial topic guide. Prior to the workshops we provided training on conducting a focus group. The community researchers invited participants, and organised the venue and catering for the event.

The workshops were attended by 15–28 men with prostate cancer, and some female family members. Community researchers facilitated small group discussions about the following topics, with a researcher observing:

- What is prostate cancer and how is it treated?
- What are your prostate cancer research priorities?
- How can we enable research participation by men from your community?
  — Sharing of broad questions about prostate cancer research to stimulate discussion: bone health in men taking androgen deprivation therapy; digital technology in prostate cancer care and follow-up.

A visual scribe presented a lay summary at the end of each workshop. The key barriers to research participation were mistrust based on widespread experience of racism, concerns about historical abuse of power, and unethical research on ethnic minority populations. The men shared their common struggle to get prostate-specific antigen testing: *‘I know more than my GPs about my high risk of prostate cancer’*, and asked, *‘How long will it take for this research to change our care?’* Additional research priorities included evaluation of a prostate cancer screening programme for high-risk populations and including black men. The community researchers and Qizhi Huang co-presented the findings at the national ‘Black in Cancer’ research conference (2022). A new third sector-funded Sheffield support group for Black African Caribbean men with prostate cancer has been developed from the work (https://www.1in4spsg.org).

## STEPS TO INCLUSIVE RESEARCH AND POWER SHARING

Achieving true citizen research power is a long-term goal: the precise steps to move from tokenism towards citizen control are unclear. We have drawn from the published literature and our public engagement work to propose the following key steps:

1. Build trust and dialogue by exchange of ideas in a community setting and led by community members.
2. Include knowledge sharing about the topic of interest with patients and communities, for example, producing lay summaries of a literature review and bringing in a topic expert for a ‘question and answer’ session.
3. Support the development of research skills in communities where it is desired (capacity building).
4. Co-creation from the outset and at every stage of the research process to include generating and prioritising research questions relevant to the public.

**Figure 2.**
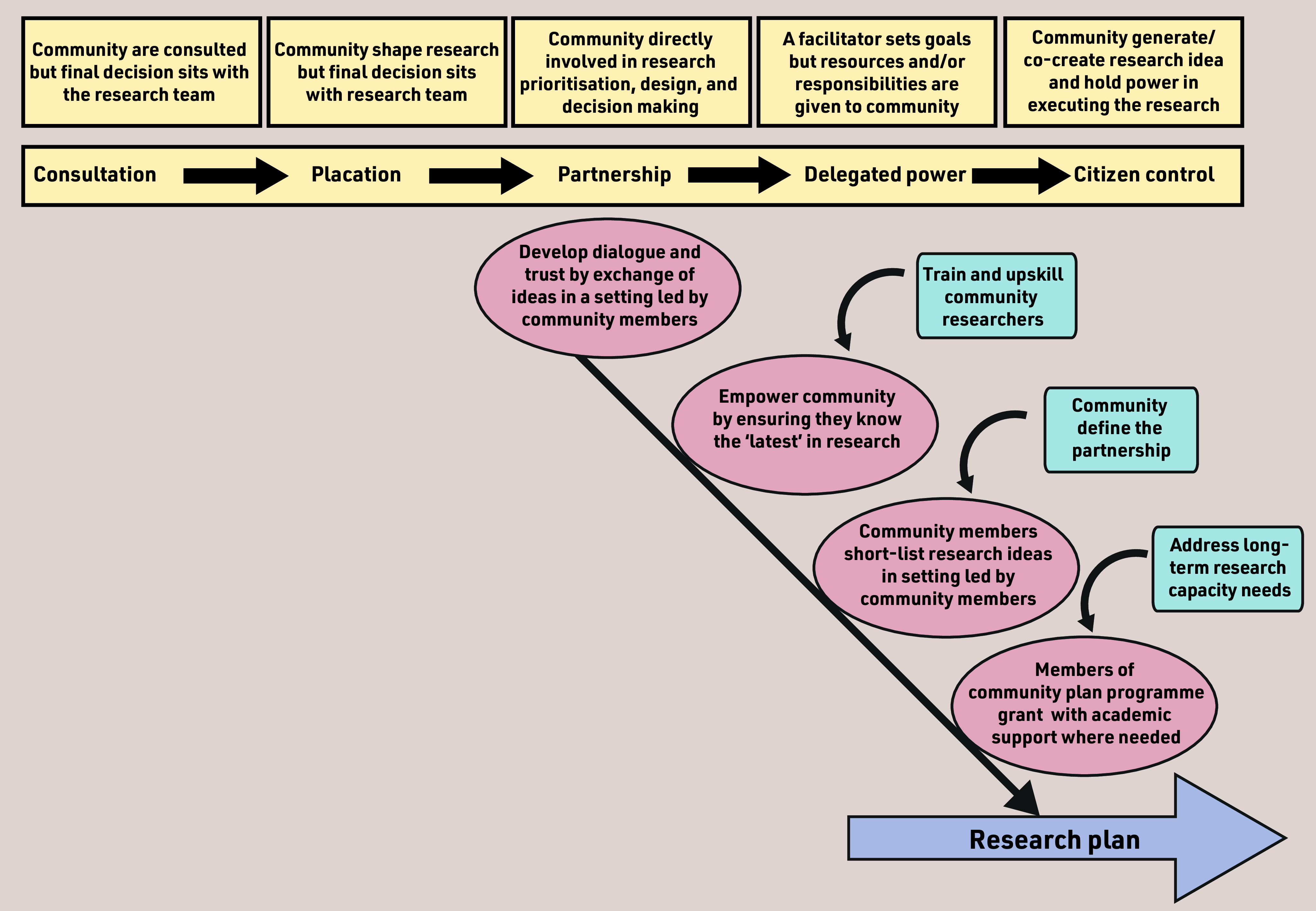
*Steps to community empowerment in the research process.*

In Figure 2, we use an example of how a researcher-led agenda might be transformed by considering power redistribution within the research process, by sharing resources, and so forging more equal partnerships between academic institutions and community groups to co-create and deliver research.

A post-COVID pandemic shift in how research is resourced, such as funded interpreters and ‘agile’ nurse support for recruitment of underserved populations, has made it more feasible to recruit participants from the DE-CRN.^19^ Community engagement is a priority in English CRNs, for example, Yorkshire and Humber have an ‘Ethnic Minority Research Inclusion’ group to drive community engagement and wider participation in research. The DE-CRN practices continue to be highly challenged by their underresourced, complex workload, but where they have actively recruited to studies have found that resources and approaches are better matched to inclusive study delivery.

Our approach to participatory inclusive research has attracted wider national interest with positive feedback from other researchers about DERA-facilitated co-creation of research with public and patients. In a study to develop a framework to address primary care inequities, the DE-PPI group were included at every stage of the research — co-interpreting evidence and data to co-produce the final framework.^20^ We recognise that the redistribution of power in primary care research requires sustained efforts. The rocky road to a powerful research partnership between patients, the public, practitioners, and researchers, who are committed to addressing health inequalities, continues at the Deep End.
